# Rapid MALDI mass spectrometry imaging for surgical pathology

**DOI:** 10.1038/s41698-019-0089-y

**Published:** 2019-07-04

**Authors:** Sankha S. Basu, Michael S. Regan, Elizabeth C. Randall, Walid M. Abdelmoula, Amanda R. Clark, Begoña Gimenez-Cassina Lopez, Dale S. Cornett, Andreas Haase, Sandro Santagata, Nathalie Y. R. Agar

**Affiliations:** 1 Department of Pathology, Brigham and Women’s Hospital, Harvard Medical School, Boston, MA 02115 USA; 2 Department of Neurosurgery, Brigham and Women’s Hospital, Harvard Medical School, Boston, MA 02115 USA; 3 Department of Radiology, Brigham and Women’s Hospital, Harvard Medical School, Boston, MA 02115 USA; 4 Bruker Daltonics, Billerica, MA 01821 USA; 5 Bruker Daltonik GmbH, Bremen, Germany; 6 Ludwig Center at Harvard, Harvard Medical School, Boston, MA 02115 USA

**Keywords:** Metabolomics, Surgical oncology, Molecular imaging, Metabolomics, Molecular medicine

## Abstract

Matrix assisted laser desorption ionization mass spectrometry imaging (MALDI MSI) is an emerging analytical technique, which generates spatially resolved proteomic and metabolomic images from tissue specimens. Conventional MALDI MSI processing and data acquisition can take over 30 min, limiting its clinical utility for intraoperative diagnostics. We present a rapid MALDI MSI method, completed under 5 min, including sample preparation and analysis, providing a workflow compatible with the clinical frozen section procedure.

## Introduction

The frozen section procedure dates back over a century^1^ and remains the primary mode of intraoperative tissue analysis in nearly every operating room in the world. Briefly, the procedure involves transport of a surgical specimen to the frozen section room, where it is rapidly frozen in embedding medium, cryosectioned, stained with hematoxylin and eosin (H&E), and microscopically assessed by an experienced pathologist.^2^ The histopathological impression is then reported to the surgeon who uses the information for surgical guidance. Despite its widespread utilization, the frozen section procedure has limitations in sensitivity, specificity, and inter-observer variability, especially in difficult cases.^3–5^ Additionally, many diseases, including most cancers, undergo early metabolic alterations prior to manifesting morphologic changes.^6,7^ As a result, the ability to perform biochemical analysis on tissue sections has been a long-held aspiration in pathology to provide clinically actionable information.

Matrix assisted laser desorption ionization mass spectrometry imaging (MALDI MSI) has emerged as a promising analytical technique capable of generating spatially resolved imaging of proteins, lipids, and small molecules in tissue specimens.^8–11^ Unlike immunohistochemical or nucleic acid-based approaches, MALDI MSI is a label free technique, which provides comprehensive and unbiased tissue characterization. To accomplish MALDI MSI, a thinly cryosectioned tissue is mounted on a metal-coated glass slide and evenly coated with an organic matrix. Next, a high-resolution scan of the slide is generated prior to loading into the MALDI source, where the sample is registered, and regions of interest for analysis are then selected. MSI data is acquired by firing a laser on the surface of the tissue, where the energy is absorbed by the matrix molecules, which are ionized along with co-crystalized biomolecules and measured using a mass analyzer, commonly a time-of flight (TOF) instrument. By performing MALDI TOF analysis in two-dimensions, the spatial distribution of thousands of molecules within the specimen can be mapped in a single analysis. This methodology has shown tremendous potential in a variety of research applications, although lengthy pre-analytical processing and acquisition times (collectively >30 min) have limited the clinical application of MALDI MSI,^12^ a problem that is particularly relevant for intraoperative diagnosis by pathologists in which time is of the essence. Here, we describe an optimization strategy to reduce the preparation and analytical time considerably, and demonstrate its performance on both mouse brain tissue and surgical resection specimens from patients.

## Results

**Fig. 1:**
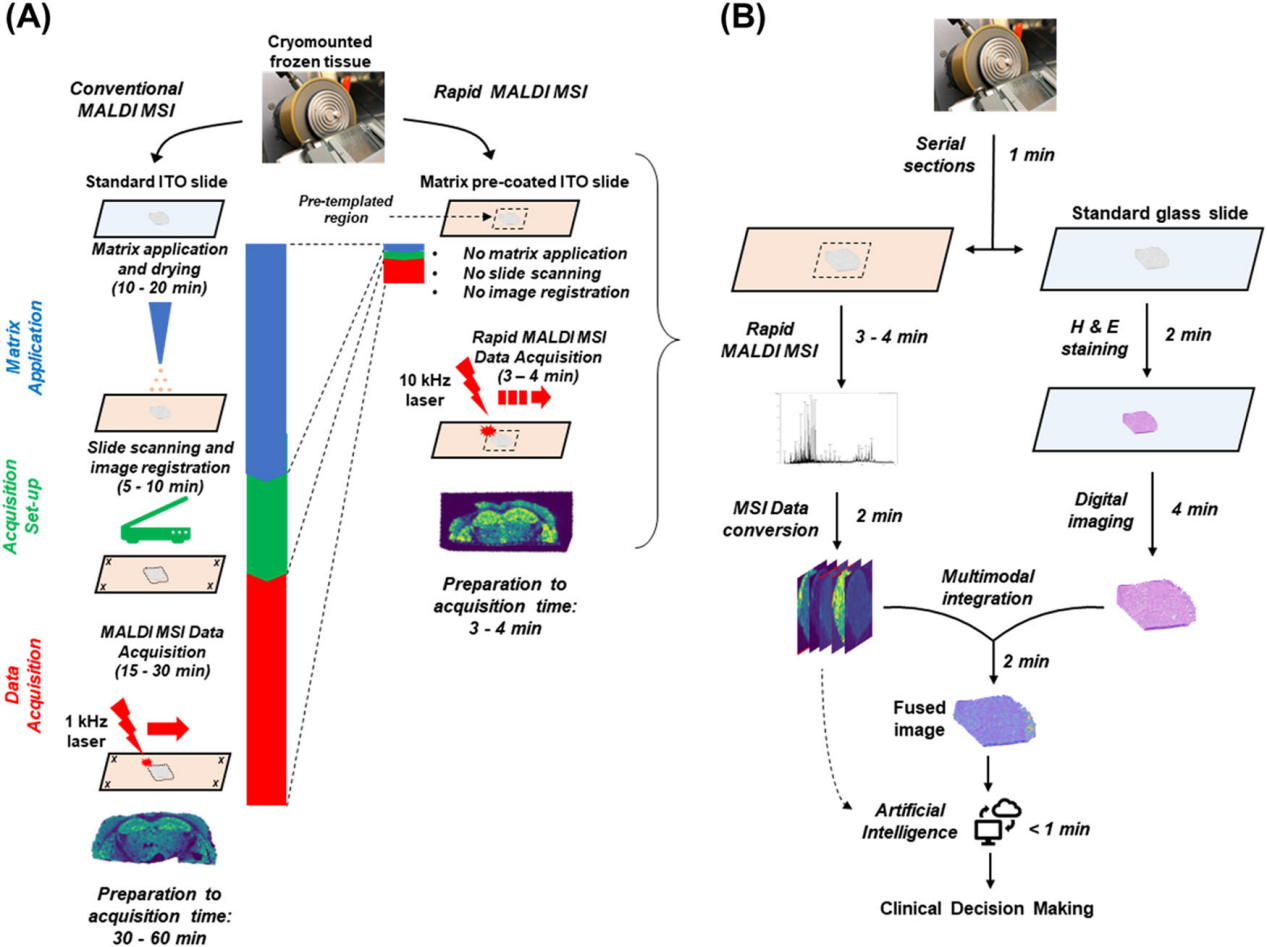
**a** Comparison of workflow for conventional versus rapid MALDI MSI. The left panel illustrates additional steps and time needed for conventional MALDI MSI, including matrix application/drying, high-resolution slide scanning, image registration, and data acquisition using a 1 kHz laser. In the rapid MALDI MSI method (right panel), by using a matrix pre-coated slide and cryomounting the sample directly into a pre-templated region, and acquiring data with a 10 kHz laser, significant improvements in turnaround time are achieved. A representative ion image (for *m/z* 885.6) is illustrated below the workflow. Additional ion images comparing conventional and rapid method can be found in Supplemental Information. **b** Proposed workflow for rapid MALDI MSI in the frozen section room. Surgical specimens are serially cryosectioned onto a glass slide and a matrix pre-coated ITO slide. The section on the glass slide is stained using H&E followed by high-speed digital imaging, while the rapid MALDI MSI method is applied to the templated region on the ITO slide in a similar time frame. Following data acquisition, either pathologist-guided or artificial intelligence (AI)-guided diagnostic approaches may be utilized. In the pathologist-guided mode, the digital histological image is fused to the ion images using a rapid non-linear transformation (Supplemental Fig. 1), allowing the pathologist to select a region or pixel of interest for further molecular characterization. This approach could be used to quickly evaluate, for example, the presence or absence of cancer or of pathogens as well as particular molecular features of the cancer cells, a feat which is currently unachievable in a similar time frame. In the AI-guided mode (dashed arrow), the MSI data could be analyzed directly without visual review using previously machine trained models to classify regions based on their mass spectral signature

The three most time-consuming steps to generate MALDI MSI are (1) *matrix application*, (2) *acquisition set-up*, and (3) *MSI data acquisition*. By shortening steps 2 and 3 and effectively eliminating step 1 (Fig. 1a), we provide here a rapid MALDI MSI method, which can be performed in <5 min including preparation and analytical time. This simple and widely implementable method could transform the current workflow of the frozen section room by providing a very powerful analytical tool for pathologists (Fig. 1b). Our optimization strategy is described in detail in the Discussion.

After achieving substantial improvements in processing time for MALDI MSI, we applied this “rapid” method to cryosectioned brains from healthy mice, which highlighted all major landmarks of the mouse brain, including the cerebral cortex, hippocampal regions, thalamus, basal ganglia, and globus pallidus. In highly myelinated regions such as the corpus callosum, we found a high relative abundance of lipids characteristic of white matter, such as *m/z* 888.7, which we identified using high-resolution mass spectrometry as a sulfatide species, ST(d42:2), a sphingolipid abundant in myelin. Conversely, in gray matter regions, we observed a strong signal for *m/z* 885.6, which we confirmed to be phosphoinositol, PI(36:4), a lipid found in abundance in higher cellular regions such as gray matter.

**Fig. 2:**
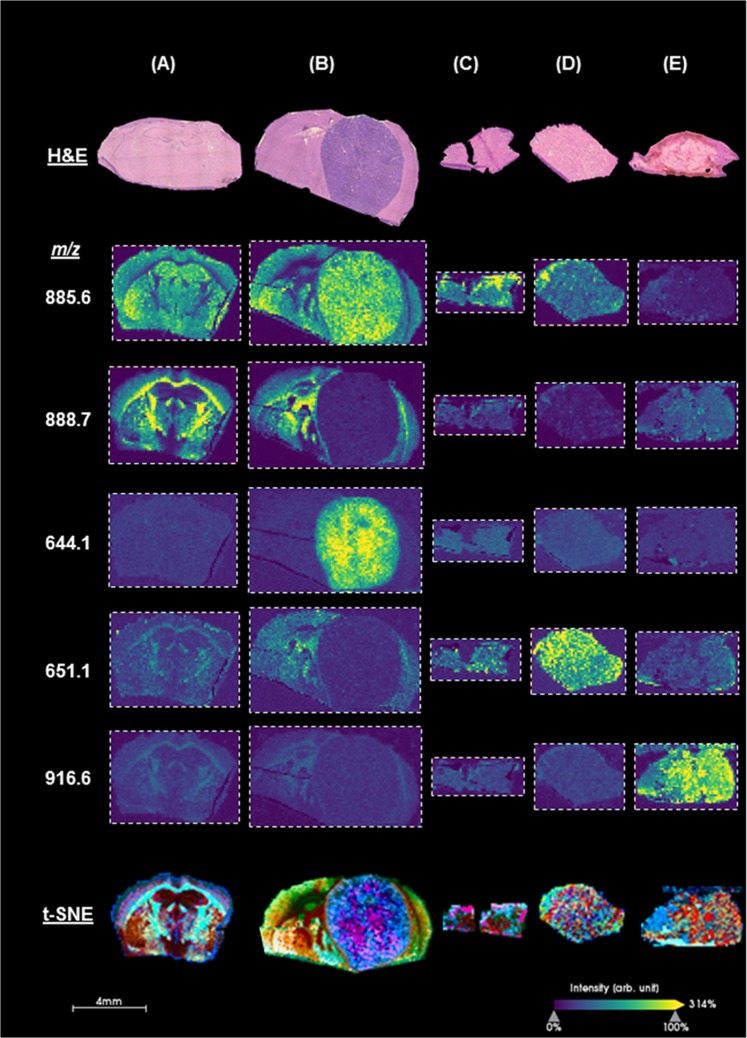
Histology and ion images generated by rapid MALDI MSI and t-distributed stochastic neighbor embedding (t-SNE) visualization of analyzed data. Coronal sections of **a** healthy mouse brain, and **b** mouse brain with a glioblastoma patient-derived xenograft. Surgically resected clinical research specimens of **c** normal breast tissue, **d** breast carcinoma, and **e** glioblastoma. The ion images were generated using SCiLs Lab software. Mass to charge ratio (*m/z*) of imaged analytes are listed on the left. Ions *m/z* 885.6 and *m/z* 888.7 represent PI(36:4) and ST(d42:2), respectively. Remaining ions are under investigation and will be identified using a combination of high-resolution mass spectrometry and fragmentation. Additional ion images are provided in the Supplemental Information, Suppl. Fig. 2

Next, we utilized this technique in a patient derived xenograft (PDX) model of glioblastoma, where we found several unique molecules, such as *m/z* 644.1, which was specifically increased in the PDX tumor (Fig. 2b). Additionally, we applied the method to surgically resected specimens collected in the Advanced Multimodality Image Guided Operating (AMIGO) suite at Brigham and Women’s Hospital. These specimens included breast cancer resection specimens that included both histologically normal regions and tumor, as well as glioma resections. As illustrated in Fig. 2c–e, we found a higher relative abundance of particular ions in both the glioma and breast tumor specimens compared to normal tissue (additional ion images are provided in SI, Suppl. Fig. 2). For each of the analyzed tissue specimens, we used t-distributed stochastic neighbor embedding (t-SNE) algorithm^13^ to reduce the high-dimensional data into 3-dimensions. We then adopted methodology^14^ to spatially project the t-SNE features onto the tissue specimen (Fig. 2, lower panel), revealing molecularly defined structures that are not apparent histologically, and thereby enhancing the diagnostic power of the frozen section procedure.

## Discussion

The first pre-analytical step we addressed was matrix application. Conventionally, spotting, spraying, or subliming of the matrix is used to coat the tissue. Spotting can result in significant analyte delocalization making it unacceptable for many imaging applications. Spraying or subliming the matrix is therefore preferred, but these steps take 15–30 min, which includes drying time. We effectively eliminated matrix application by mounting tissue sections on indium tin oxide (ITO) slides that we pre-coated with matrix.^15^ As we were most interested in negative mode analysis, we used 2′,4′,6′-trihydroxyacetophenone monohydrate (THAP) as a matrix and cryosectioned tissue at 5-micron thickness. A single coat of THAP was optimal. Multiple coats prevented proper mounting and adhering of the tissue sections to the pre-coated slide, and, moreover, the additional coats of matrix did not significantly improve signal intensity (see SI, Suppl. Fig. 3).

The next significant time constraint in MALDI MSI is setting-up data acquisition. This step begins with generating a high-resolution scan of the mounted tissue. The slide is then loaded into the instrument and mapped using designated “teach points”, which are used for image registration. Next, the user defines a region of interest (ROI) on the slide on which to perform MALDI MSI. High-resolution scanning, image registration, and ROI selection together can consume 10–15 min per slide. To shorten this interval, we used a templated ITO slide with a pre-delineated region for analysis programmed in a standard auto executable run file. By mounting the sample into this pre-registered region, we were able to analyze the specimen without prior scanning. The pre-templated region is large enough to accommodate and easily mount a frozen surgical specimen, though small enough to maintain a short analytical run time. A region measuring 1.2 × 0.6 cm was sufficient for most clinical specimens and could be analyzed in <3 min when imaged at a 100-micron spatial resolution. If higher spatial resolution is required, a smaller targeted region can be re-analyzed at 20–50 micron resolution.

The last time-consuming step we addressed was the MALDI MSI data acquisition itself, which is largely determined by the laser frequency, spatial resolution and the number of laser shots per pixel. Recent technological improvements have substantially reduced analytical time by using higher frequency lasers.^16–18^ We capitalized on the high-frequency laser of the rapifleX MALDI Tissuetyper (Bruker Daltonics, Billerica, MA), which is capable of firing up to 10 kHz, and employs a novel rastering movement and software improvements, which decreases image acquisition time by a factor of 10x–20x compared to previously available MALDI TOF platforms. The laser firing pattern had a significant impact on ion intensity and image quality, and we found that the *M5 flat* pattern, which defines a focused laser spot of ~75 µm and nearly uniform fluence, provided the highest quality mass spectrometry images. Subsequent examination of the analyzed slide using reflective bright-field microscopy confirmed greater penetration and tissue ablation using this pattern likely contributing to the improved imaging (SI, Suppl. Figs. 4–6). After optimization, we achieved a combined preparation and run time—from sectioning to MALDI MSI acquisition—of <5 min. In terms of the clinical workflow, these data could then be analyzed either directly using machine learning approaches or mapped to H&E stained sections to provide a rich complementary data set and tool to aid in histopathological analysis.

This rapid MALDI MSI method achieves a total preparation and run time of under 5 min and is thereby fully compatible with the time constraints of frozen section histological analysis. This innovation opens the door to the ultimate goal of chemical imaging in the frozen section room to complement histopathology and advance the diagnosis of human disease and patient care.

## Methods

### Chemicals and materials

HPLC grade water and acetonitrile (ACN) were obtained from Fisher Scientific (Pittsburgh, PA). 2′,4′,6′-trihydroxyacetophenone monohydrate (THAP) was obtained from Sigma-Aldrich (St. Louis, MO). Optimal cutting temperature (OCT) compound was obtained from Thermo Fisher Scientific (Carlsbad, CA). Conductive indium tin oxide (ITO) coated glass slides were obtained from Bruker Daltonics (Billerica, MA).

### Generation of matrix pre-coated slides

To prepare pre-coated slides, THAP was dissolved in 9:1 ACN: water (v/v) at a concentration of 40 mg/mL and sonicated for 10 min in a sonicator bath (Fisher Scientific, Pittsburgh, PA). The matrix solution was then applied to an ITO slide using a TM Sprayer (HTX Technologies, Chapel Hill, NC) at 30 °C, flow rate of 0.05 mL/min, velocity of 1200 mm/min and track spacing of 1.5 mm. Varying numbers of matrix coatings (passes) were tested, but a single coat was found to be optimal, and was therefore used for all subsequent analyses. The gas pressure of the nitrogen supplied to the sprayer was set to 10 psi with a gas flow rate of 2 L/min, and the nozzle height set to 40 mm with a 2 s dry time.

### Tissue preparation/application

Animals studies were conducted according to protocols approved by the Institutional Animal Care and Use Committee at the Mayo Clinic. Flash frozen whole brains from female Harlan Sprague Dawley Athymic Nude-Foxn1nu (Envigo, Indianapolis, IN) were kindly provided by the laboratory of Dr. Jann Sarkaria (Mayo Medical, Rochester, MN). Prior to analysis, brains were equilibrated to −20 °C, affixed to the chuck using a small amount of OCT and coronally cryosectioned at 5 µm thickness using a HM550 cryostat (Thermo Fisher Scientific, Carlsbad, CA) and thaw mounted onto the THAP pre-coated ITO slides. For surgical specimen analysis, normal and breast tissue specimens as well as glioma specimens were acquired intraoperatively in the Advanced Multimodality Image Guided Operating (AMIGO) suite at the Brigham and Women’s Hospital (Boston, MA). Research subjects gave written informed consent to the Dana-Farber Cancer Institute Institutional Review Board (IRB) protocols (DFCI 10–417 and DFCI 14–476). Tissue sectioning and slide preparation of the clinical specimens was performed in a similar fashion and using the same parameters as done for the mouse brains.

### Rapid MALDI MSI analysis

Rapid MALDI MSI was performed using a rapifleX MALDI Tissuetyper (Bruker Daltonics, Billerica, MA). FlexControl (Bruker Daltonics, Version 4.0) was used to optimize and acquire data using the following parameters: negative ion polarity, mass scan range (*m/z* 360–1000), digitizer (1.25 GHz), sample rate (0.8 ns), detector voltage (2795 V), spatial resolution at 100 µm, 50 shots per pixel, and 10 kHz laser frequency. Real time smoothing was turned off, analog offset (70.2 mV), sensitivity of detector (100 mV/full scale), and laser beam focus set to 25. Matrix suppression was set to deflection for any *m/z* <287, PIE delay (100 ns), ion source 1 and 2 set to 20, and 17.28 kV, respectively. Lens voltage (11 kV) while reflector voltage 1, 2, and 3 (20.76, 1.085, 8.6 kV). “Height Detect” parameters were turned off to reduce pre-analytical time. The region for analysis was set using FlexImaging software (Bruker Daltonics, version 5.0), and individual images were visualized using both FlexImaging and SCiLS Lab (Bruker Daltonics). All ions in acquired spectra were quantified and represented as a percentage of total ion current (TIC). Spatially resolved ions identified using MALDI TOF were subsequently confirmed using a MALDI FT ICR (9.4 T Solarix, Bruker Daltonics).

### High-dimensional data visualization

The dimensionality reduction method of t-distributed stochastic neighbor embedding (t-SNE) was used to non-linearly map the high-dimensional data points (i.e., spectra) into a reduced subspace of three-dimensional (3D) representation. Briefly, t-SNE computes pairwise similarities of high dimensional data points as such similar data points are projected close to each other into the lower dimensional representation while dissimilar data points are projected further away. We adopted the methodology proposed by Abdelmoula et al.^14^ to spatially map those 3D t-SNE features in which each of the t-SNE coordinates was spatially organized and fed into a separate color channel in L*a*b* color system. The separation distances of dissimilar points in the scatter space translate into edge structures in the image space. The spatially mapped t-SNE features form a composite image, called t-SNE image, that represents the entire MSI datacube and reveals molecular distinct structures.

### High-dimensional multi-modal data integration

We used t-SNE-based image registration methods^19,20^ to enable automated multi-modal integration between MSI and H&E stained images. In this approach, the t-SNE image structurally enriches to establish spatial correspondences with the H&E image and thus enables the non-linear registration. We first performed an affine transformation, which captures global deformations (e.g., translation, rotation, scaling, and shear) and then linearly mapped the t-SNE image to the same coordinate space as the H&E image. We then used the non-linear transformation model of cubic B-Spline to capture local deformations (e.g., compression). For fast convergence and minimizing the likelihood of local minima, we implemented the non-linear registration process in a multi-resolution scheme using four resolution levels of Gaussian smoothing pyramids.^21^ The transformation model was optimized using the stochastic gradient descent optimizer^22^ and the registration quality was assessed using the statistical metric of mutual information.^23^ The non-linear image registration method was implemented using the publicly available registration toolbox of elastix.^21^ The t-SNE image was then non-linearly warped to be spatially aligned with the H&E, and the optimized transformation matrix was applied on each *m/z* image to be integrated with the histological structures.

## Supplementary information

Supplementary Information

## Data Availability

The data that support the findings of this study are available from the corresponding author upon reasonable request.

## Supplementary information

**Supplementary information** accompanies the paper on the *npj Precision Oncology* website (10.1038/s41698-019-0089-y).

## Code availability

The computer codes used in this study are available from the corresponding author upon reasonable request.
