# Supplementary material for: Rapid MALDI mass spectrometry imaging for surgical pathology
Source: NPJ Precis Oncol. 2019 Jul 4;3:17. doi: 10.1038/s41698-019-0089-y (PMC6609678; doi:10.1038/s41698-019-0089-y)
Supplement: Supplementary file 1 — Supplementary Information [file 41698_2019_89_MOESM1_ESM.pdf]

# Supplementary Information

## *Rapid MALDI Mass Spectrometry Imaging for Surgical Pathology*

**Authors:** Sankha S. Basu[1€], Michael S. Regan[2€], Elizabeth C. Randall[3], Walid M. Abdelmoula[2], Amanda R. Clark[2], Begoña Gimenez-Cassina Lopez[2], Dale S. Cornett[4], Andreas Haase[5], Sandro Santagata[1,6], and Nathalie Y.R. Agar[2,3]

**Affiliations:**

[1] Department of Pathology, Brigham and Women's Hospital, Harvard Medical School, Boston, Massachusetts 02115, United States

[2] Department of Neurosurgery, Brigham and Women's Hospital, Harvard Medical School, Boston, Massachusetts 02115, United States

[3] Department of Radiology, Brigham and Women's Hospital, Harvard Medical School, Boston, Massachusetts 02115, United States

[4] Bruker Daltonics, Billerica, MA 01821, United States

[5] Bruker Daltonik, Bremen, Germany

[6] Ludwig Center at Harvard, Harvard Medical School, Boston, Massachusetts 02115, United States

[€] *S.S.B. and M.S.R. contributed equally to this work*

***Corresponding Author***

*Mailing Address: Nathalie Y.R. Agar, Ph.D., Hale Building for Transformative Medicine, 60 Fenwood Road, Suite 8016-J, Boston, Massachusetts 02115, United States; Tel: 617.525.7374; Fax: 617.264.6316; E-mail: Nathalie_Agar@dfci.harvard.edu. ORCID Nathalie Y. R. Agar: 0000-0003-3149-3146.

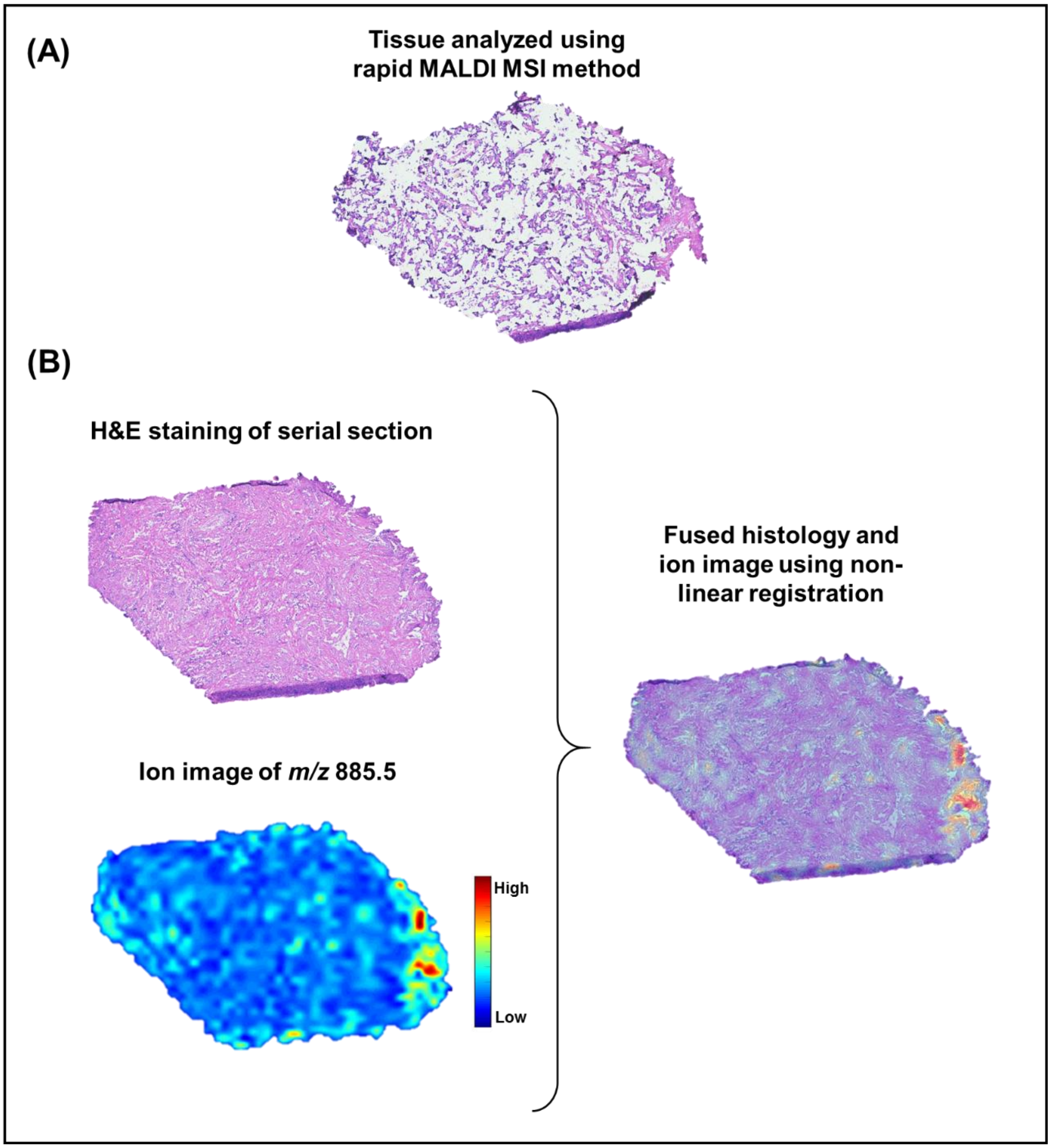


Supplemental Figure 1. Histology and non-linear ion image fusion. (A) H&E staining of breast cancer tissue after rapid MALDI-MSI analysis demonstrating significant tissue destruction during MSI data acquisition, making post-hoc histological analysis currently unfeasible. (B) To correlate histological features with MSI data, H&E can be performed on a serial section and fused with ion images from the MALDI MSI analyzed specimen using a non-linear registration approach. This approach would allow a pathologist to either select a particular region with morphological features of interest for further metabolomic interrogation, or to perform more detailed histopathological examination of regions which demonstrate biochemical profiles characteristic of a disease feature, an infectious pathogen or another lesion of interest.

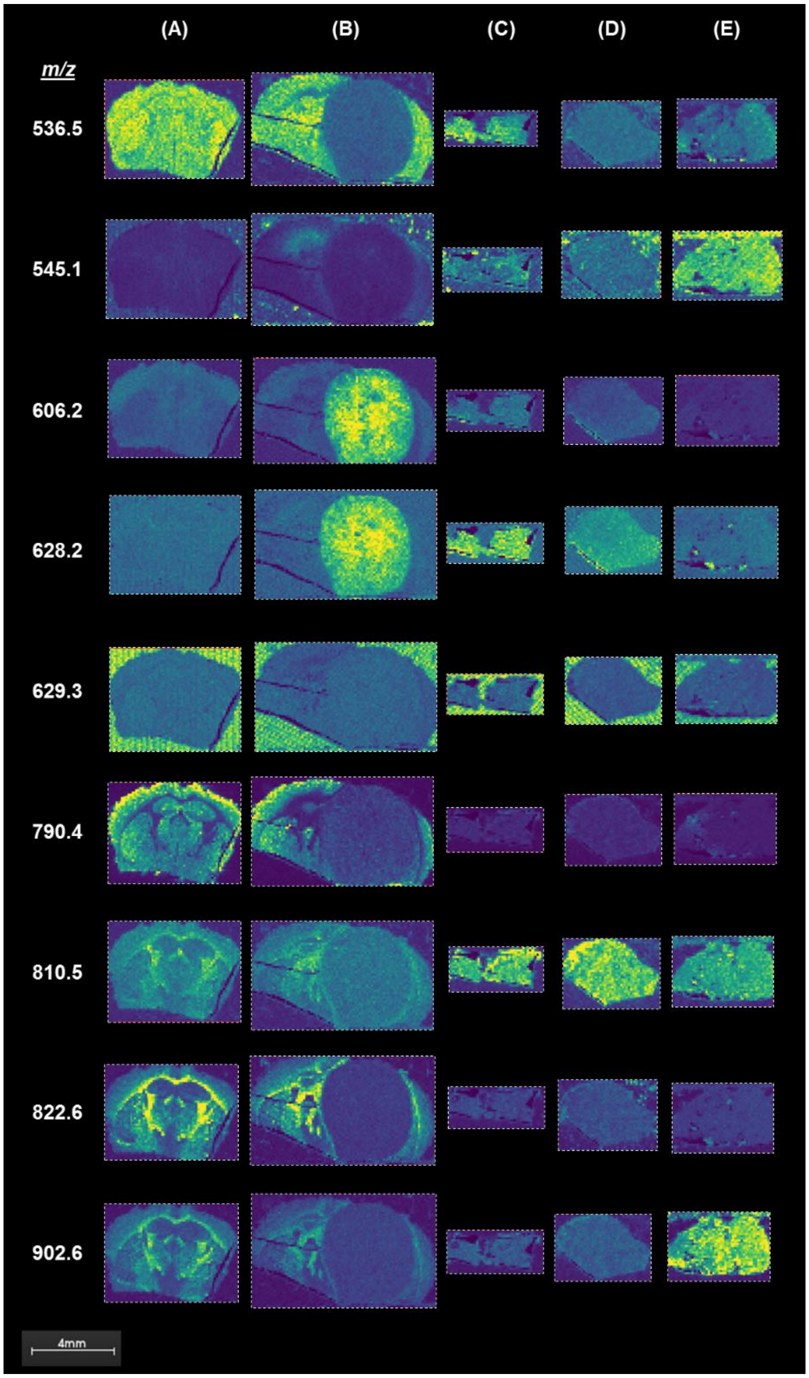


Supplemental Figure 2. Additional ion images generated using the rapid MALDI MSI method on cryosectioned tissue specimens: (A) normal mouse brain, (B) mouse brain from a PDX model of glioblastoma, (C) grossly normal region of breast lumpectomy, (D) tumor region from the breast lumpectomy and (E) human glioma specimen from the tumor core. The mass to charge ratio (*m/z*) presented in the images is on left.

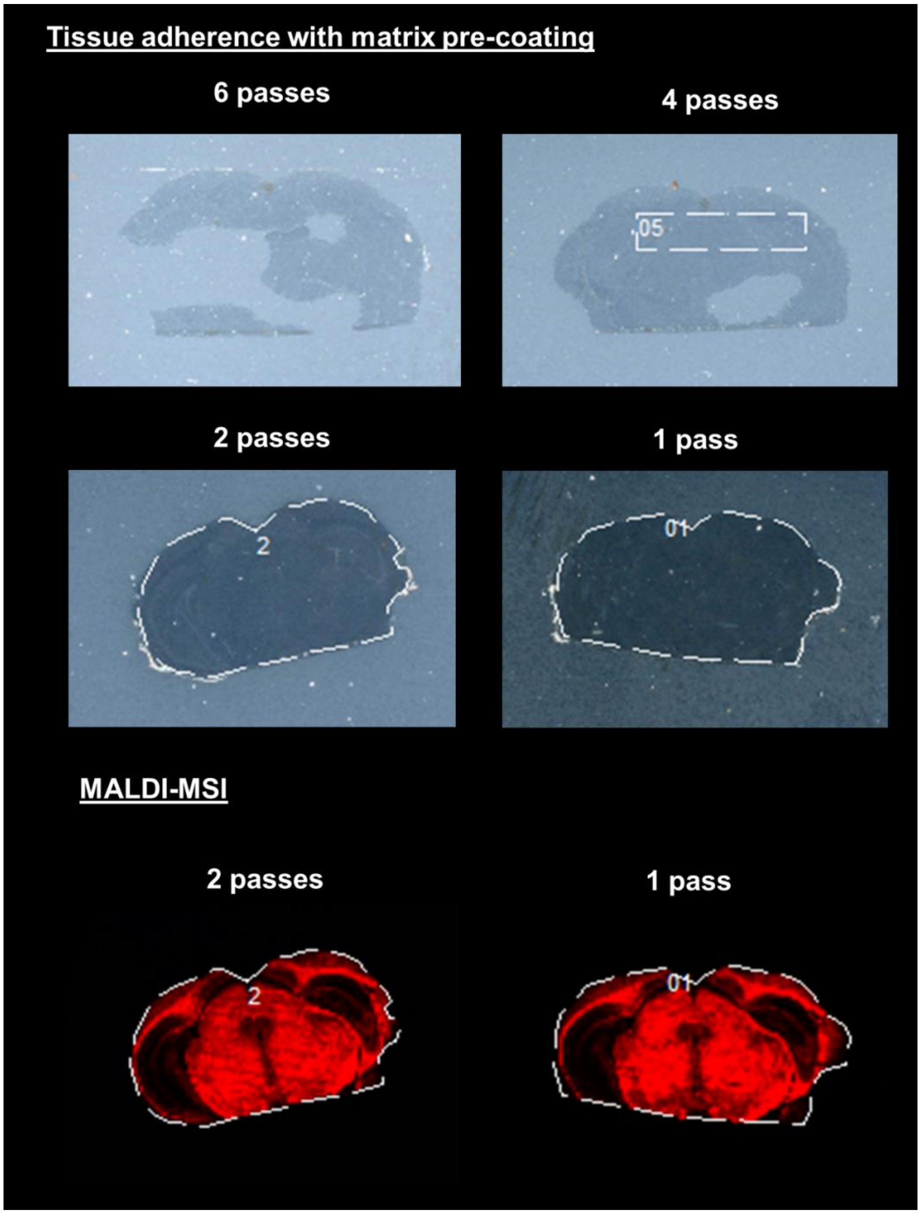


Supplemental Figure 3. Scanned images of sectioned tissue on pre-coated slides demonstrating effect of multiple passes of THAP matrix on tissue adherence. Bottom panels show correlative MALDI MSI from specimens mounted on one or two coats of matrix.

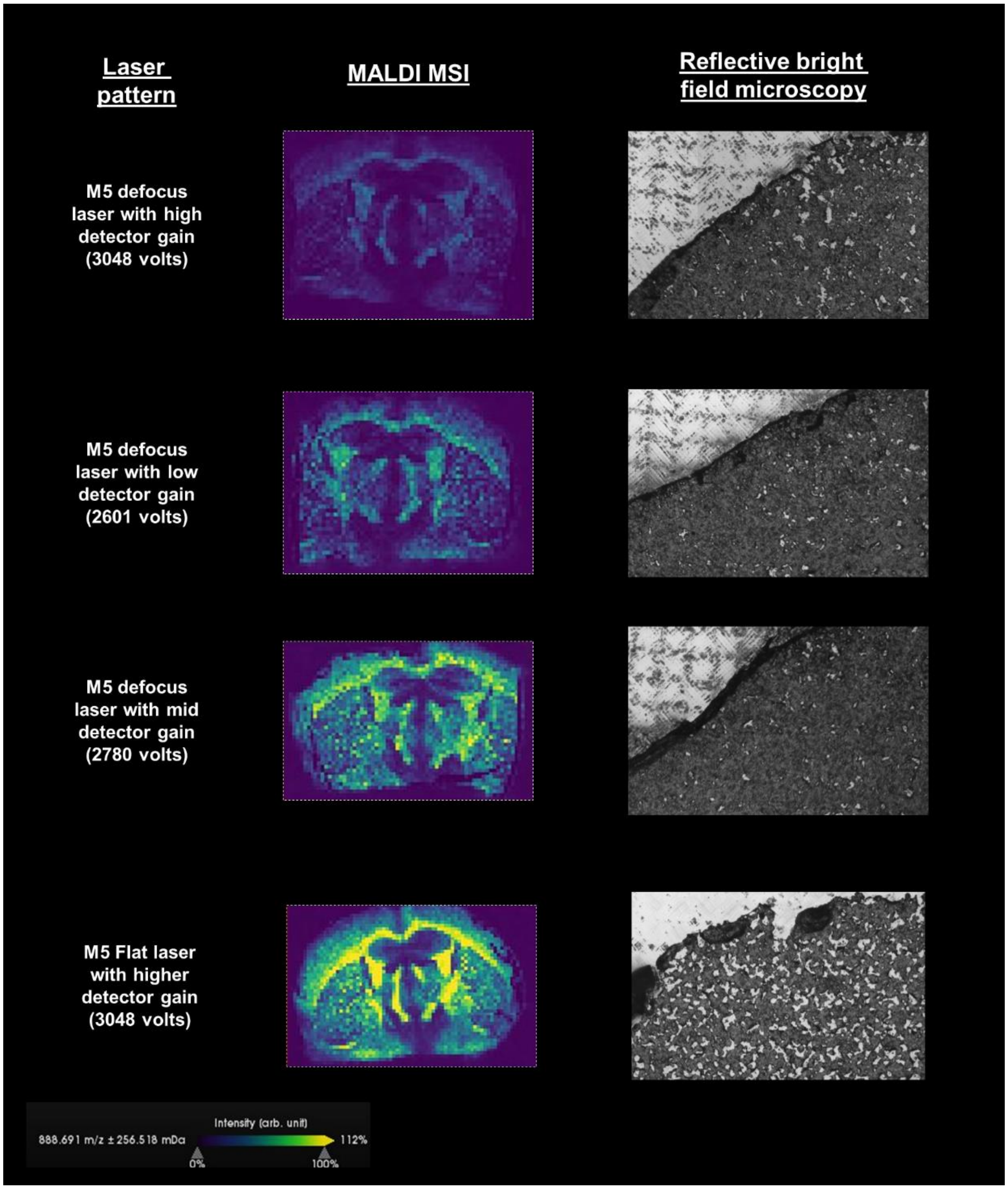


Supplemental Figure 4. MALDI MSI and reflective bright field microscopy of mouse brain tissue post-analysis using different laser firing patterns. Ion images of *m/z* 888.7, ST(d42:2), a sulfatide abundant in myelinated structures, are represented on the left images next to the laser firing pattern and detector gain used. The M5 Flat laser demonstrated the most reproducible signal and allowed the most clear visualization of neuroanatomical structures. Reflective bright field microscopy of the tissue analyzed using various laser firing patterns, demonstrates more complete tissue ablation using the M5 flat laser pattern. The higher signal intensity could be attributed to the matrix being located below the tissue (as opposed to above the tissue as is done in conventional MALDI MSI) and therefore benefits from increased penetration through the tissue.

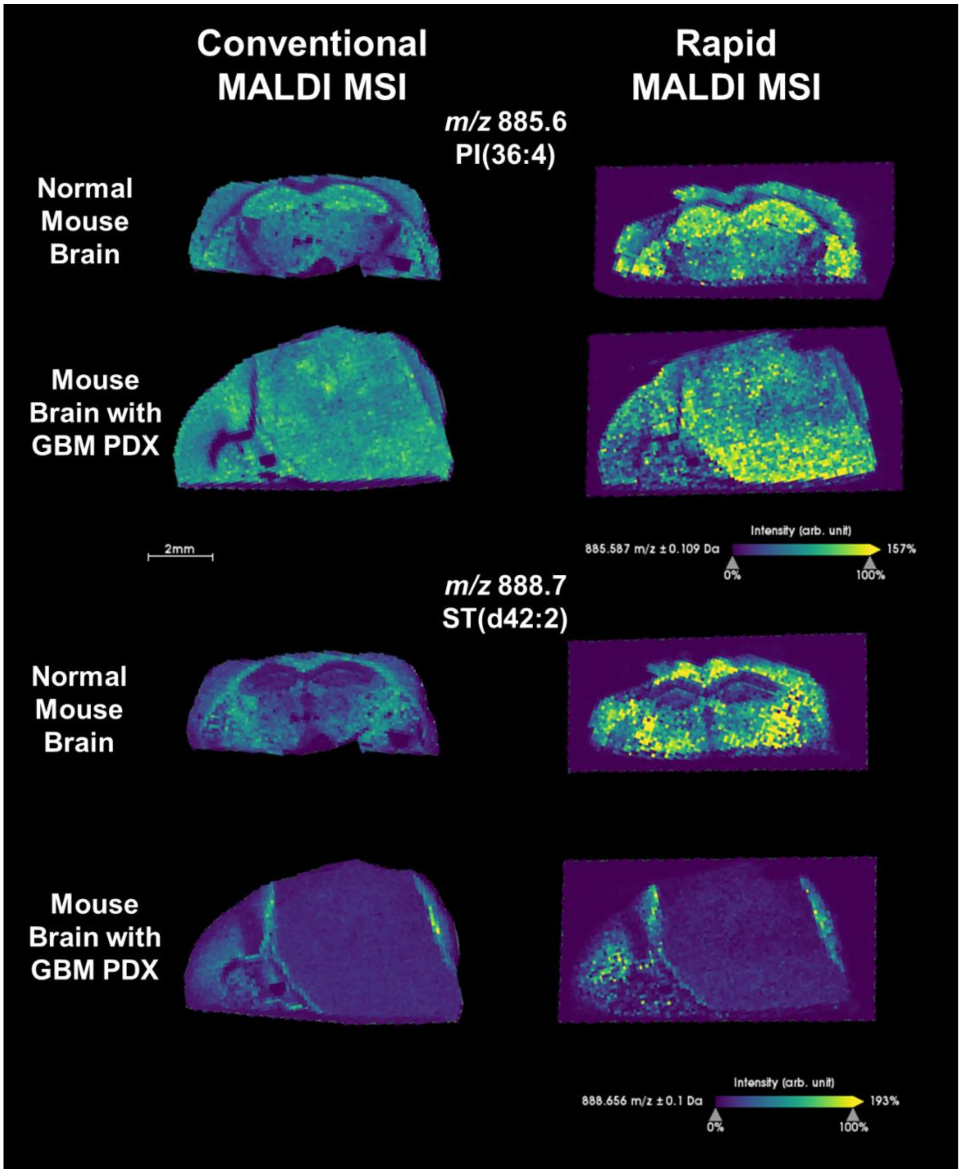


Supplemental Figure 5. Ion images for m/z 885.6 (PI 36:4) and 888.7 (ST d42:2) generated using either conventional or rapid MALDI MSI method on cryosectioned normal mouse brains and mouse brains containing tumors in a PDX model of glioblastoma. As demonstrated in the right panel, there was significantly higher signal intensity in the rapid MALDI MSI images.

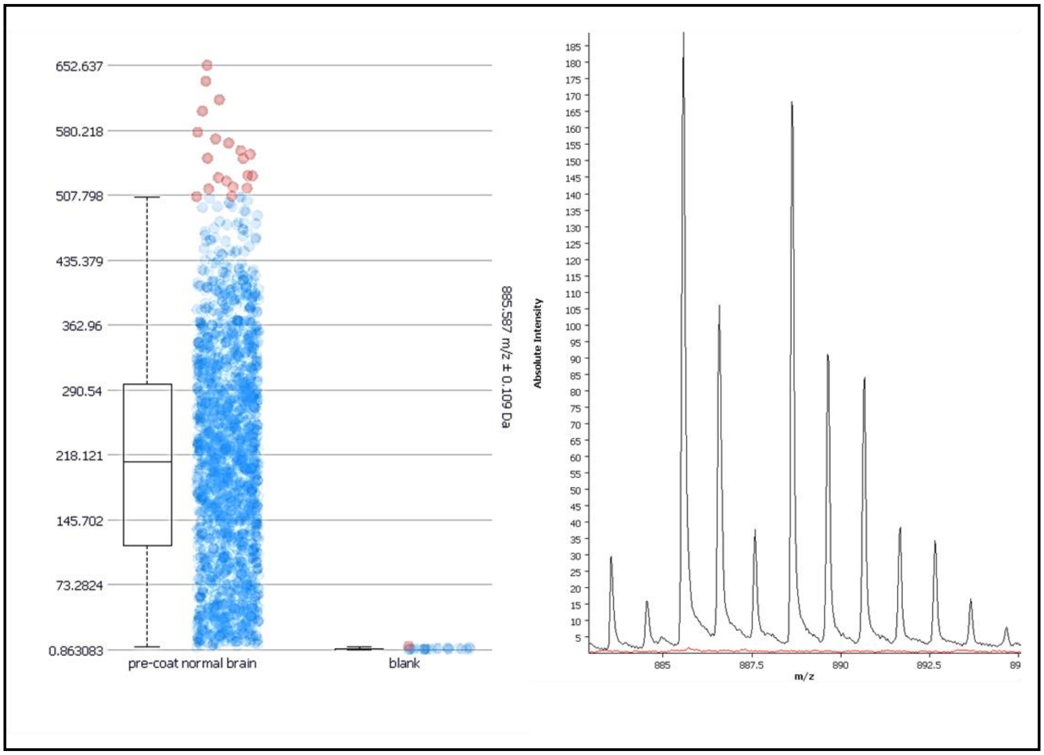


Supplemental Figure 6. Signal to noise ratio. On the left, is a box blot showing the relative ion intensity of *m/z* 885.6, PI (36:4), comparing pixels containing tissue to blank pixels coated with matrix alone. On the right is a cumulative ion spectra from these regions (black line represents tissue regions, and red line demonstrates blank regions).
